# Alexithymia as a Bridge Between Negative Affect and Schizotypy: A Cross‐Sectional Network Model

**DOI:** 10.1002/cpp.70156

**Published:** 2025-09-19

**Authors:** Ercan Ozdemir, Angus MacBeth, Helen Griffiths

**Affiliations:** ^1^ School of Health in Social Science University of Edinburgh Edinburgh UK

**Keywords:** alexithymia, emotion regulation, negative affect, network modelling, schizotypy

## Abstract

**Background:**

Schizotypy provides a theoretically grounded framework for investigating psychosis proneness, reflecting a multidimensional structure that parallels the positive, negative and disorganized symptoms of schizophrenia. Alexithymia, a trait characterized by difficulties in identifying (DIF) and describing feelings (DDF) and a tendency towards externally oriented thinking (EOT), has been robustly linked to schizophrenia. Despite these associations, the relationship between alexithymia and schizotypy remains underexplored. Moreover, given that both constructs are closely associated with negative affect, it is critical to account for this potential confound to estimate their associations accurately.

**Method:**

We employed a cross‐sectional design in a non‐clinical sample to examine the associations among the dimensions of alexithymia, negative affect and schizotypy. Network models were estimated using Spearman correlations and the EBICglasso method to capture conditionally independent associations.

**Results:**

The sample (*N* = 420) was predominantly female (*n* = 314), with ages ranging from 18 to 37 years (*M* = 28.72, SD = 4.52). More than half (*n* = 232) reported receiving mental health treatment, and 127 participants self‐reported a personal history of psychosis. DIF and negative schizotypy emerged as central to different clusters in the network. Specifically, DIF served as a bridge linking positive and disorganized schizotypy dimensions to anxiety and stress, whereas negative schizotypy was a central bridge connecting DDF and EOT to depression. These estimates were psychometrically stable.

**Conclusion:**

Our findings suggest DIF and negative schizotypy as plausible mechanisms of change facilitating emotional attunement and resilience against distress due to unusual self‐experiences.

## Summary

- Difficulties in identifying feelings may signal elevated risk for psychotic‐like experiences, particularly disorganized thoughts, perceptual aberrations and magical ideation.
- Negative schizotypy may be central in sustaining the associations between depression, externally oriented thinking and difficulties in describing feelings.
- Longitudinal methods are required for a robust examination of the interactions between the stress‐reactive cluster and the negative schizotypy‐driven cluster.
- Individual differences in stability and change in the identified clusters may inform personalized intervention methods.

## 1 Introduction

Schizotypy has traditionally been conceptualized as a latent trait indicative of vulnerability to schizophrenia, with cognitive slippage, marked by disorganized thinking, representing its fundamental feature (Meehl 1962, 1990). This model posits cognitive slippage as central to psychosis risk, while anhedonia and interpersonal aversiveness intensify clinical severity and underpin poor prognosis. Contemporary research has redefined schizotypy as a multidimensional construct, comprising positive (magical ideation and perceptual aberrations), negative (anhedonia and emotional detachment) and disorganized (akin to cognitive slippage) dimensions that parallel the symptom structure of schizophrenia (Cohen et al. 2015; Kwapil and Barrantes‐Vidal 2015; Lenzenweger 2006). Of note, the multidimensional schizotypy framework does not assume any centrality among the schizotypy dimensions. Clarifying the structure and interplay of these dimensions is essential for improving the identification of at‐risk individuals, refining early intervention strategies and advancing transdiagnostic models of psychosis risk.

The schizotypy dimensions appear consistent across the psychosis continuum, from latent traits of schizotypy to high‐risk states to full‐blown psychosis (Fonseca Pedrero and Debbané 2017; Nelson et al. 2013; Rosell et al. 2014), but may also manifest as transdiagnostic phenomena (Linscott and van Os 2013; van Os and Reininghaus 2016). The disorganization dimension is associated with various mental health conditions, including depression, mania (Hart and Lewine 2017; Sass and Parnas 2017) and borderline personality disorder (Kwapil et al. 2022). Similarly, social anhedonia, a defining quality of the negative dimension, is commonly observed in individuals diagnosed with schizophrenia, depression (Krynicki et al. 2018), post‐traumatic stress disorder, eating disorders and autism spectrum disorders (Barkus and Badcock 2019). These findings underscore the importance of examining how schizotypy dimensions interact with each other and with transdiagnostic symptoms and risk factors (Linscott and van Os 2013; van Os and Linscott 2012).

Reframing schizotypy within a network theoretical framework offers a complementary perspective to dimensional models by conceptualizing schizotypy dimensions not as passive expressions of an underlying trait but as interactive elements of a broader psychological system (Borsboom 2017). From a network theoretical perspective, schizotypal disorganization may function as a central component activating and maintaining other components, such as social anhedonia or magical ideation. This reconceptualization can be supported by psychometric network models identifying schizotypal disorganization as the most central schizotypy dimension (Dodell‐Feder et al. 2019; Hasson‐Ohayon et al. 2018), potentially mediating the relationship between negative and positive dimensions (Christensen et al. 2019). Emerging evidence further suggests that disorganization, rather than positive or negative schizotypy, may uniquely predict heightened stress sensitivity and the exacerbation of psychotic experiences in daily life, pointing to its role as a critical determinant of psychotic reactivity to stress (Hernández et al. 2023; Kemp et al. 2023; Rónai et al. 2025). These findings underscore the need to investigate the relationship between disorganization and affective dynamics to better understand its contribution to stress‐related psychological disorders.

Alexithymia is a multidimensional construct implicated in the maintenance of maladaptive affective dynamics and is denoted by difficulties in identifying (DIF) and describing feelings (DDF), alongside a limited capacity for imagination and a tendency towards externally oriented thinking (EOT) (Taylor and Bagby 2013). Alexithymia dimensions can be conceptualized as cooperative components collectively hindering emotion processing (Taylor et al. 2024). For example, alexithymia may represent a process, in which the inability to identify feelings may impair emotion regulation by hindering processing and describing emotions (e.g., Jurist 2005), while EOT may further contribute to DIF through a pronounced tendency to avoid reflective engagement with internal states (e.g., Preece et al. 2017). Evidence suggests that alexithymia is a transdiagnostic factor prevalent in eating disorders (Nowakowski et al. 2013), depression (Li et al. 2015), substance use (Honkalampi et al. 2022) and suicide ideation (Hemming et al. 2019), as well as psychotic disorders (O'Driscoll et al. 2014; Ozdemir, Xiao, et al. 2025). However, research examining alexithymia in schizophrenia and psychosis risk predominantly involves case–control comparisons of alexithymia as a unidimensional construct limiting the understanding of synergistic interactions among its dimensions and their differential associations with symptom domains and underlying affective dynamics (Ozdemir, Xiao, et al. 2025).

Research investigating the multidimensional relationships between alexithymia and psychosis risk suggests that DIF is consistently associated with the positive dimension from high‐risk states (Fung et al. 2017; van Rijn et al. 2011; Yang et al. 2020) to clinical symptomatology (Maggini and Raballo 2004; Yu et al. 2011). In contrast, negative schizotypy appears more robustly linked to alexithymia, showing stronger associations with DIF, DDF and EOT (Larøi et al. 2008; Martin et al. 2016). Importantly, DIF may not only reflect a stable trait‐like characteristic but also emerge as a reaction to stress (Luminet et al. 2021), and alexithymic features may be elevated by persistent depressive and anxious states (Taylor and Bagby 2013). In this context, schizotypal disorganization may further disrupt emotional awareness and contribute to affective reactivity.

Meta‐analytic evidence reports moderate positive associations between disorganized schizotypy and both DIF and DDF, though these effects did not reach statistical significance due to the limited number of studies (*k* = 3) (Ozdemir, Xiao, et al. 2025). Furthermore, the review highlights the possibility that these associations may be inflated due to insufficient control for confounding variables, particularly negative affect. Emerging evidence from a longitudinal network model suggests a developmental cascade in which elevated schizotypal disorganization predicts increased mental state uncertainty (analogous to DIF) 9 months later, which in turn predicts elevations in positive schizotypy (Ozdemir, MacBeth, and Griffiths 2025). Notably, mental state uncertainty also functioned as a concurrent bridge, linking anxiety and stress to disorganized schizotypy, highlighting its role as a transdiagnostic mechanism activating the feedback between negative affect and cognitive‐perceptual disruptions.

To address these limitations and advance our understanding of how specific schizotypy and alexithymia dimensions coactivate within an affective network, the present study implements a cross‐sectional network modelling approach. Unlike traditional analyses that often rely on latent constructs or assume unidirectional pathways, network analysis offers a data‐driven framework that captures direct relationships among observed variables while controlling for all other variables in the network (Borsboom and Cramer 2013). This allows for the simultaneous examination of multiple interdependencies, revealing how symptoms cluster, reinforce one another or serve as bridges between domains (Jones et al. 2021). Network modelling thus provides a unique method to identify bridging factors, key variables that link distinct symptom dimensions, shedding light on potential mechanisms of comorbidity and informing intervention strategies.

In this study, we aim to (1) map the network organization among alexithymia dimensions (DIF, DDF and EOT), schizotypy dimensions (positive, negative and disorganized) and transdiagnostic affective symptoms (depression, anxiety and stress) and (2) identify bridging components that spread the coactivation of maladaptive factors relevant to psychosis risk. We hypothesize two distinct yet interrelated clusters: (a) a cluster involving DIF, anxiety, stress and positive schizotypy, the connectivity of which is maintained by DIF, and (b) a cluster involving negative schizotypy, depression, DDF and EOT. By modelling these dynamics, we aim to delineate how distinct constellations of traits and affective processes may cooperate to maintain psychosis risk.

## 2 Methods

### 2.1 Sample

Participants were drawn from a two‐wave longitudinal cohort study; the present analysis focuses on the 9‐month follow‐up (Wave 2), comprising a cross‐sectional subsample of *n* = 420 participants. Recruitment for the follow‐up occurred between July and September 2022, with invitations sent to 1105 individuals who had completed the baseline survey. The baseline sample was initially recruited via online advertisements targeting the general population. To ensure the relevance of assessed experiences, the advertisements were designed to reach individuals with a range of psychological distress or emotional difficulties, using broadly targeted mental health‐related keywords.

Inclusion criteria required participants to be between 18 and 35 years old and fluent in English. As such, the sample was restricted to residents of English‐speaking countries (Australia, Canada, Ireland, New Zealand, South Africa, the United Kingdom and the United States). Full details of the recruitment procedures and baseline characteristics are reported in a previous publication (Ozdemir et al. 2024).

Participants with incomplete data on the follow‐up survey were excluded. Missing data followed a systematic pattern, primarily at the scale level, as the survey was structured in blocks. This likely reflects participant burden over the ~30‐min completion time. Outliers were retained, with Spearman correlations used in the network analysis to mitigate their influence.

### 2.2 Measures

A demographic questionnaire collected information on participants' age, gender and included mental health questions about the participant's lifetime history of psychosis (i.e., Have you ever experienced a psychotic episode?), current treatment for mental health issues (i.e., Are you currently receiving mental health treatment?) and any history of psychosis in their immediate family (i.e., Does anyone from your immediate family have a diagnosis of a psychotic disorder?). The mental health questions were responded to categorically with the options ‘Yes’, ‘No’ and ‘Prefer not to say’.

#### 2.2.1 Alexithymia

The Toronto Alexithymia Scale (TAS; Bagby et al. 1994) is a three‐dimensional self‐report measure of alexithymia assessing DIF and DDF and EOT. A psychometric concern arises with respect to the reliability of the EOT subscale, which has exhibited inconsistent metrics in psychometric studies involving translated versions of the TAS (Bagby et al. 2020), with cross‐cultural variance undermining its generalizability. Moreover, construct validity concerns arise from meta‐analyses of alexithymia across diverse clinical populations revealing weak or non‐significant associations between the EOT subscale and depression (Li et al. 2015), suicide (Hemming et al. 2019), self‐harm (Norman et al. 2020), substance use (Honkalampi et al. 2022) and eating disorders (Nowakowski et al. 2013).

Despite these psychometric concerns, it is important to recognize that EOT remains a core dimension of the alexithymia construct, indicating a mode of operative thinking that is disengaged from inner experience and symbolic elaboration (Taylor et al. 2023). Notably, meta‐analytic findings indicate that EOT demonstrates moderate positive associations with schizophrenia diagnosis (Ozdemir, Xiao, et al. 2025), supporting its relevance in the context of schizotypy research, even as its associations with other clinical phenomena remain weak or inconsistent. In light of the foundational role of EOT in the alexithymia construct and the emerging evidence for its clinical significance, we retained the EOT dimension in our network model. To address psychometric limitations, we employed a reduced, three‐item EOT subscale, the structure of which was guided by a psychometric procedure involving exploratory and confirmatory factor analyses (refer to the Supporting Information for a detailed account of the reduced measurement model). In the current study, DIF (*α* = 0.86), DDF (*α* = 0.83) and the three‐item EOT (*α* = 0.70) subscales' reliability metrics were acceptable.

#### 2.2.2 Negative Affect

The Depression Anxiety Stress Scale‐21 (DASS; Antony et al. 1998) is a self‐report measure of negative affect rated on a 4‐point scale. The depression, anxiety and stress scales include seven items in each dimension. Psychometric studies of the DASS support its reliability and validity (Brown et al. 1997; Zanon et al. 2021). The depression (*α* = 0.91), anxiety (*α* = 0.85) and stress (*α* = 0.87) dimensions of the DASS showed acceptable reliability in the current study and were included in the network models.

#### 2.2.3 Schizotypy

Multidimensional Schizotypy Scale‐Brief (MSS; Gross et al. 2018) measured the negative, disorganized and positive dimensions of schizotypy with 38 items rated on a categorical response scale. The negative schizotypy dimension involved 13 items assessing social anhedonia and diminished emotional experiences. The disorganized schizotypy dimension is a 12‐item index of incoherence in cognition and behaviour, whereas positive schizotypy was measured with 13 items indicating magical ideation and perceptual aberrations. The MSS shows acceptable convergent validity based on correlations with observer‐rated schizotypy scales (Kemp et al. 2020). The reliability estimates for negative (*α* = 0.84), disorganized (*α* = 0.92) and positive dimensions (*α* = 0.84) were acceptable, and all three dimensions were included in the network models.

#### 2.2.4 Aberrant Salience

The Aberrant Salience Inventory (ASI; Cicero et al. 2010) is a self‐report assessment of psychosis‐near experiences with 29 items rated on a dichotomous scale. The ASI was used in this study to psychometrically identify psychosis proneness based on the cut‐off score of > 13.5 applied to the total scale score (Merola et al. 2023). Based on this criterion, psychosis‐prone experiences are well represented in our sample, with 241 individuals (57.4%) scoring above the threshold. The unidimensional ASI showed acceptable reliability (*α* = 0.93).

### 2.3 Data Analysis

All analyses were conducted using RStudio (Version 2023.06.1; Posit Team 2023). Descriptive statistics were computed to examine sample characteristics. Network analyses were performed to estimate the cross‐sectional associations among the alexithymia, negative affect and schizotypy dimensions.

Network models were estimated using the bootnet package (Epskamp and Fried 2018), employing the extended Bayesian information criterion graphical least absolute shrinkage and selection operator (EBICglasso) estimator (Tibshirani 1996). This approach applies regularization to estimate sparse networks by shrinking small edge weights to zero, thereby improving model interpretability and reducing the risk of overfitting. Given the presence of non‐normally distributed variables, the network estimation was based on Spearman rank‐order correlations.

The network model included nine nodes, referring to network representations of the study variables, which are dimensions of alexithymia (DIF, DDF and EOT), negative affect (depression, anxiety and stress) and schizotypy (negative, positive and disorganized). The network involved 36 possible undirected edges, each representing a regularized partial correlation between two nodes, controlling for all other nodes in the network. Visualization of the networks and computation of edge weights were conducted using the qgraph package (Epskamp et al. 2012).

Centrality indices were estimated to evaluate the structural role of each node. Specifically, strength centrality was defined as the sum of the absolute edge weights connected to a node, indicating the overall level of direct connectivity. Bridge centrality, estimated using the networktools package (Jones 2022), quantifies the degree to which a node serves as a connector between distinct network communities, calculated as the sum of absolute edge weights linking a node to nodes in other clusters. Nodes exhibiting high bridge strength facilitate the transmission of influence or activation across otherwise distinct psychological domains.

The reliability of the estimates was evaluated using the ‘bootnet’ package (Epskamp et al. 2018). Edge weight accuracy was estimated using a case‐drop bootstrap procedure, and subset bootstrapping was applied to estimate the correlation stability coefficient (CSC). A CSC value below 0.25 indicates unacceptable stability of the centrality estimates (Epskamp et al. 2018). Given that recommended sample sizes for stable estimation of edge weights in sparse cross‐sectional networks with fewer than 20 nodes range from 250 to 350 (Constantin and Cramer 2022), the adequacy of the sample size was also considered when interpreting the results.

## 3 Results

### 3.1 Sample Characteristics

Participants who provided complete research data were included in the analysis. The sample (*n* = 420) self‐identified as 75% female, 13% male and 12% non‐binary, and participants were aged between 18 and 37 years (*M* = 28.72, SD = 4.52). The sample included *n* = 232 participants under mental health treatment, *n* = 127 participants with a self‐reported history of psychosis and *n* = 70 with a self‐reported psychosis diagnosis in the immediate family. In terms of employment status, *n* = 275 participants were employed, *n* = 77 participants were students and *n* = 68 participants were unemployed. Gender differences were observed, in which non‐binary participants, compared to male or female participants, reported significantly higher positive and disorganized schizotypy and DIF and DDF (see Table S1).

### 3.2 Network Models

**FIGURE 1:**
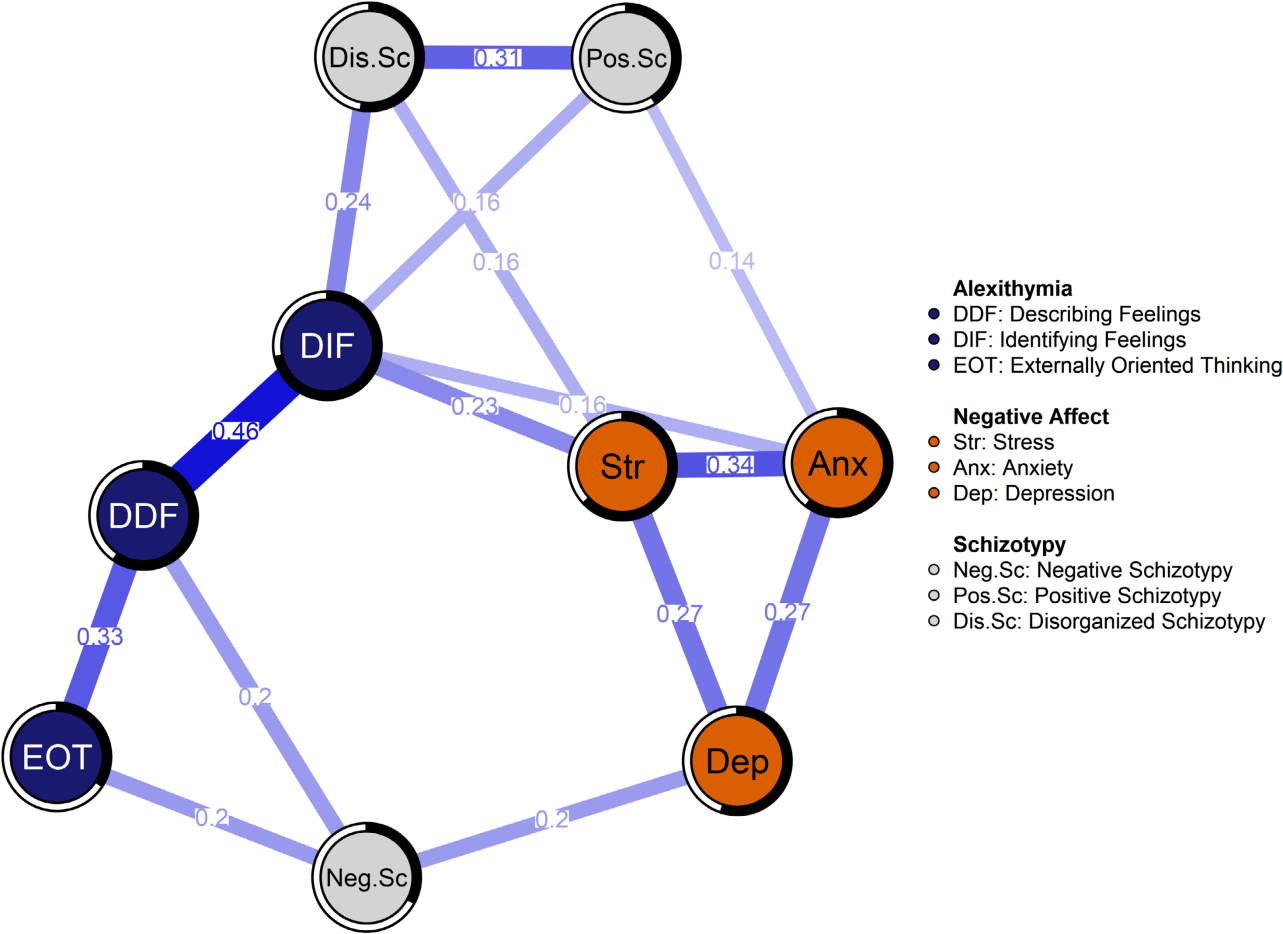
Network model of alexithymia, negative affect and schizotypy.

**FIGURE 2:**
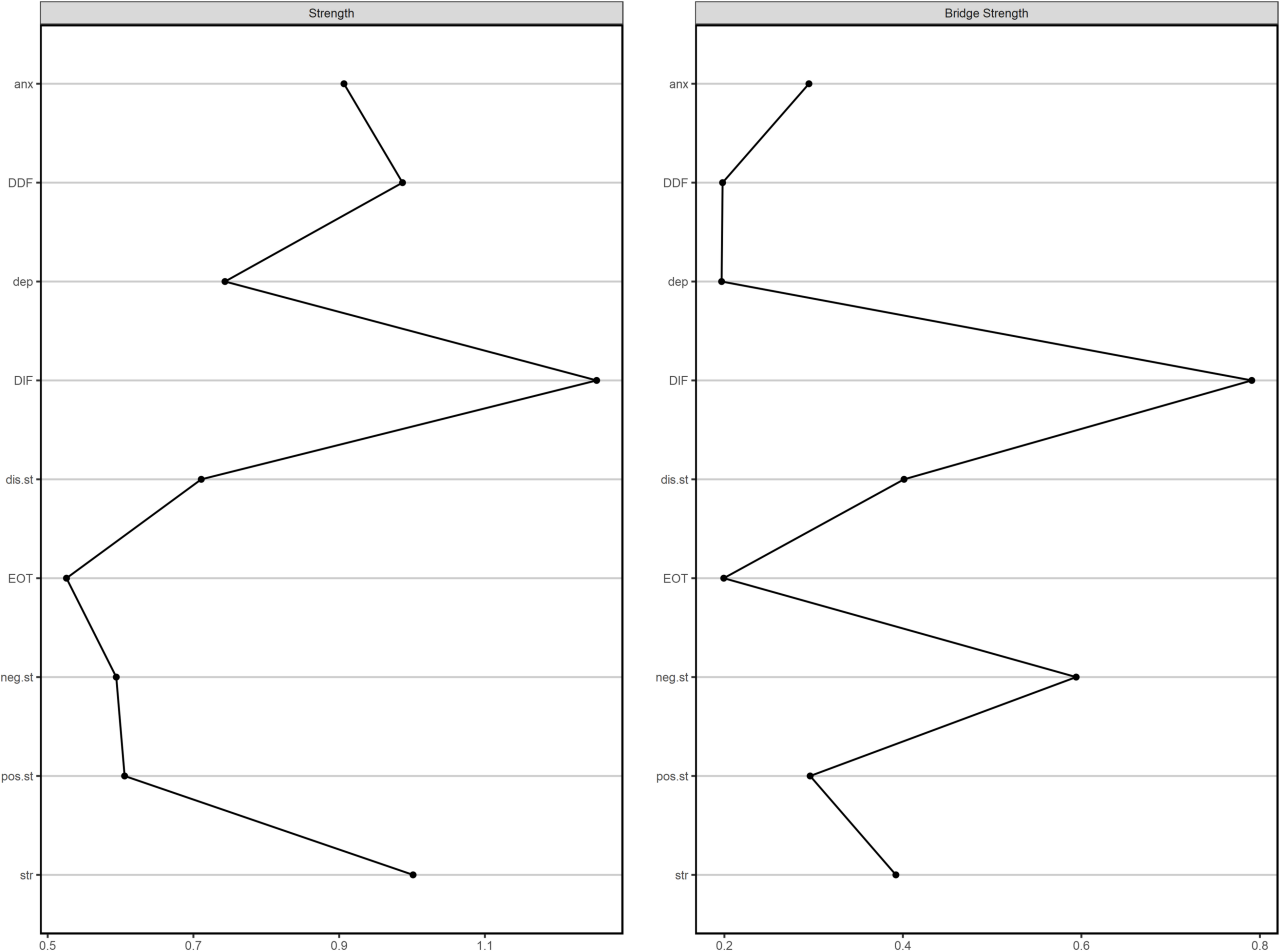
Strength and bridge strength centrality indices.

The estimated network model based on Spearman's rank‐order correlations is presented in Figure 1. Edge thickness reflects the strength of associations between nodes, with only statistically significant edges (*p* < 0.05) displayed. A complete list of significant edge weights is provided in Table S2. The network structure was moderately dense, with 42% of all possible edges observed. The centrality indices are presented visually in Figure 2 with the actual values listed in Table S3. DIF emerged as the most central node in terms of the strength of its associations with other nodes and a central bridging node connecting stress and anxiety to disorganized schizotypy and positive schizotypy. Negative schizotypy formed a second prominent bridge within the network, linking together depression, EOT and DDF. These findings imply that DIF may facilitate the transmission of stress and anxiety into broader schizotypal traits involving disorganization, magical ideation and perceptual aberrations, while negative schizotypy may perpetuate diminished emotional engagement and limited self‐disclosure.

### 3.3 Network Stability and Accuracy

The results of the non‐parametric bootstraps with 5000 samples indicated wide and overlapping confidence intervals for the edge weights, suggesting variability in edge estimates across bootstrap samples (see Figure S1 for accuracy analyses plots). Case‐drop bootstrapping with 5000 samples suggested acceptable stability for strength centrality estimates (CS‐coefficient = 0.67, 95% CI [0.60, 0.75]) and bridge strength centrality (CS‐coefficient = 0.52, 95% CI [0.44, 0.60]).

## 4 Discussion

Our study aimed to delineate the network structure of the relationships between alexithymia, negative affect and schizotypy. We hypothesized a specific grouping of the network components under two clusters. Accordingly, DIF was postulated to be central to the reinforcement of the relationships between stress, anxiety, DIF and positive schizotypy, while negative schizotypy, depression, DDF and EOT were hypothesized to be clustered together. The estimated network model supports the hypothesized clustering among the network components and DIF as a central bridge in the cluster characterized by negative arousal and unusual self‐experiences. We further identified negative schizotypy as a central bridge connecting depression to DDF and EOT. In summary, the findings suggest DIF and negative schizotypy as putative mechanisms of change fostering resilience against stress‐reactive cognitive disorganization and facilitate emotional attunement and reflective engagement.

A granular examination of the delineated associations within the observed clusters may inform about their distinct roles in the emergence and persistence of unusual self‐experiences and negative schizotypy‐driven processes. Within this framework, DIF emerges as a plausible mechanism that contributes to heightened distress in the context of schizotypal disorganization. DIF may compromise one's capacity to interpret internal emotional cues, thereby amplifying a sense of ambiguity in self‐experience, which can prompt compensatory efforts to impose coherence and meaning on uncertainty (Torre and Lieberman 2018). Idiosyncratic or unusual interpretations of experience, characteristic of positive schizotypy, might emerge from this drive to resolve uncertainty. Theoretical models of psychosis risk posit that such disorganized attempts to resolve uncertainty and distress may be a proximal indicator of impending psychosis (Kapur 2003). Similarly, a longitudinal network model identifies uncertainty about mental states as a predictor of psychosis‐near experiences 9 months later (Ozdemir, MacBeth, and Griffiths 2025). The resulting state of inability to identify feelings may subsequently foster unusual belief formation and perceptual anomalies characteristic of positive schizotypy, underscoring a mechanistic pathway through which disorganized traits contribute to unfolding psychosis vulnerability.

The negative schizotypy‐bridged cluster delineates a potential pathway through which alexithymic traits (EOT and DDF) may contribute to depressive outcomes. Previous studies have established bivariate associations between negative schizotypy and depression (Edwards et al. 2019) and difficulties in describing emotions (Larøi et al. 2008; Martin et al. 2016; Prince and Berenbaum 1993), yet these pairwise associations fail to capture their codependency. Our network results provide a more integrated view, suggesting that alexithymic traits may exacerbate depressive symptoms through their bridging role with negative schizotypy.

Poor emotional awareness and expression can foster interpersonal withdrawal and affective flattening, core features of negative schizotypy, which in turn impair emotion regulation and obstruct the formation of meaningful relational ties. Within this framework, negative schizotypy may mediate the impact of alexithymic vulnerabilities, channelling them into depression. Importantly, individual differences in the presentation and temporal stability of negative schizotypy are critical to its clinical interpretation. While persistent negative schizotypal traits may represent markers for the ontogeny of a psychosis‐proneness trajectory, with low comorbidity of depression (Bucci and Galderisi 2017), more transient presentations may reflect a response to depressive or psychotic states (Kirkpatrick 2014; Mosolov and Yaltonskaya 2022). Persistent forms are further associated with poor insight (Kirkpatrick 2014) and greater functional impairment (Devoe et al. 2021). In this context, EOT may serve as a maintaining mechanism for diminished insight, while DDF may inhibit the relational processes necessary for corrective emotional experiences and psychological integration (Taylor 2000; Taylor et al. 2023).

Limitations of the current study relate to a number of methodological issues. Although the psychometric reliability of the strength centrality and bridge centrality indices was acceptable, there remains a risk of a sampling error due to the underrepresentation of men and non‐binary individuals in the sample. This is an important limitation considering sex differences pertaining to more severe negative symptomatic presentation in men (Giordano et al. 2021), which may be associated with proximal psychosis risk indicators compared to women (Ozdemir et al. 2024).

Psychometric properties of the measurement model of alexithymia also warrant further attention. The reliability and validity of the DIF and EOT dimensions of the TAS have been questioned in previous research, with some arguing that the DIF subscale may capture general psychological distress rather than reflective capacity (Lane et al. 2015; Marchesi et al. 2014; Preece et al. 2024). The TAS has been considered as a relatively stable measure, meaning that its scores may fluctuate under conditions of heightened negative affect (Taylor and Bagby 2013). However, these fluctuations may hold clinical significance, as stress‐induced declines in reflective capacities could play a role in the acute onset of psychosis (Liotti and Gumley 2008). Supporting this, relational distress (Nolte et al. 2013) and heightened psychotic disorganization (Bora et al. 2009; Sprong et al. 2007) can compromise the ability to reflect on mental states. To better understand these interactive processes, experience sampling method offers a promising approach to examine how stress‐reactive impairments in self‐reflection contribute to the severity and persistence of psychotic experiences in daily life.

The EOT subscale presents notable psychometric limitations, including inconsistent reliability across cultural contexts (Bagby et al. 2020) and weak or non‐significant associations with key clinical outcomes such as depression, self‐harm, eating disorders and substance use (Hemming et al. 2019; Honkalampi et al. 2022; Li et al. 2015; Norman et al. 2020; Nowakowski et al. 2013). Despite these limitations, its clinical relevance warrants renewed attention. EOT reflects operative thinking, marked by the detachment of mental representations from bodily and affective experiences and an excessive focus on what is overtly observable (Taylor et al. 2023). Operative thinking may underpin disruptions in emotional processing and interpersonal attunement, positioning EOT as a potentially important marker for affective and psychosomatic vulnerability. To address psychometric concerns, we applied a factor‐analytic refinement, yielding a shortened version with improved internal consistency while preserving theoretical coherence. These findings support revisiting the EOT measurement model to better reflect its clinically meaningful features.

## 5 Conclusion

This study foregrounds the significance of DIF and negative schizotypy within a network of alexithymia, negative affect and schizotypy. Disruptions in self‐reflective processes are identified as a link between unusual self‐experiences and distress, while negative schizotypy bridged emotional and reflective disengagement to depressive affect. Thus, fostering resiliency to reflective capacity against distress could improve the management of unusual self‐experiences such as disorganization, magical ideation and perceptual aberrations. These dynamic interactions should be further explored using longitudinal designs, which could offer deeper insights into the causal pathways to psychosis development.

## Conflicts of Interest

The authors declare no conflicts of interest.

## Supporting information


**Table S1:** Pairwise comparisons of study variables by gender with Bonferroni‐adjusted Wilcoxon tests and effect sizes.
**Table S2:** Estimated edge weights of the network model.
**Table S3:** Strength and bridge centrality metrics.
**Figure S1:** Edge weight accuracy based on case‐drop bootstrapping.

## Data Availability

The data that support the findings of this study are available from the corresponding author upon reasonable request.
