# Supplementary material for: Alexithymia as a Bridge Between Negative Affect and Schizotypy: A Cross‐Sectional Network Model
Source: Clin Psychol Psychother. 2025 Sep 19;32(5):e70156. doi: 10.1002/cpp.70156 (PMC12447372; doi:10.1002/cpp.70156)
Supplement: Supplementary file 1 — Table S1: Pairwise comparisons of study variables by gender with Bonferroni‐adjusted Wilcoxon tests and effect sizes. Table S2: Estimated edge weights of the network model. Table S3: Strength and bridge centrality metrics. Figure S1: Edge weight accuracy based on case‐drop bootstrapping. [file CPP-32-e70156-s001.pdf]

Supplementary Materials

Alexithymia as a Bridge Between Negative Affect and Schizotypy: A Cross-Sectional Network Model

Ercan Ozdemir*[1] , Angus MacBeth[1], Helen Griffiths[1]

[1] School of Health in Social Science, University of Edinburgh, Edinburgh, UK

Measurement model for the Externally Oriented Thinking Dimension of the TAS

To evaluate the psychometric properties of the Externally Oriented Thinking (EOT) subscale, we employed a multi-step measurement modeling approach.

Analysis Plan

1. **Exploratory Factor Analysis (EFA):**

We conducted an EFA on the EOT items from the Toronto Alexithymia Scale (TAS-20) to assess the dimensionality of the EOT subscale. Based on EFA results (e.g., factor loadings, cross-loadings, and item-total correlations), we identified and removed poorly performing EOT items to improve internal consistency and construct validity.

2. **Confirmatory Factor Analysis (CFA) on Reduced EOT:**

The reduced EOT item set was submitted to CFA to evaluate its unidimensionality and model fit, using established fit indices (CFI ≥ 0.95, RMSEA ≤ 0.06, SRMR ≤ 0.08).

3. **CFA of Reduced EOT within the Full TAS 3-Factor Model:**

We then estimated a CFA model for the full TAS, with the reduced EOT subscale alongside the other two TAS factors (DIF, DDF) to assess the fit of the model with the revised EOT.

4. **Comparative Psychometric Evaluation:**

Model fit indices from the revised measurement models were compared to those obtained from a CFA of the original TAS-20 model, to evaluate whether the reduced EOT structure yields superior or comparable psychometric properties.

Results

1. **Exploratory Factor Analysis (EFA):**

A parallel analysis conducted on the EOT subscale indicated that two factors should be extracted. Accordingly, an exploratory factor analysis (EFA) with two factors was performed using oblimin rotation. The results showed that TAS_10, TAS_15, and TAS_19 loaded substantially on the first factor (loadings = .73, .58, and .72, respectively), while TAS_5 loaded strongly on the second factor (loading = .999). Other items did not reach the threshold for meaningful loading (≥ .30) on either factor. The first and second factors accounted for 19.0% and 13.9% of the variance, respectively, with a cumulative variance explained of 32.9%. These results suggest a multidimensional structure for the EOT subscale, with distinct item groupings across two factors.

2. **Confirmatory Factor Analysis (CFA) on Reduced EOT:**

A confirmatory factor analysis (CFA) was conducted to evaluate the fit of a unidimensional measurement model for the EOT subscale, comprising TAS_10, TAS_15, and TAS_19 as indicators. The model was estimated using the WLSMV estimator appropriate for ordinal data. Fit indices were not interpreted due to the model being just-identified with only three items; by definition, such models will always exhibit perfect fit. Instead, the focus is placed on the standardized factor loadings, which were substantial for all three items (TAS_10 = .78,

TAS_15 = .60, TAS_19 = .77), providing support for the unidimensionality and internal consistency of the reduced EOT subscale.

3. **CFA of Reduced EOT within the Full TAS 3-Factor Model:**

A confirmatory factor analysis (CFA) was conducted to evaluate the fit of a three-factor model of the TAS, in which items loaded on Difficulty Identifying Feelings (DIF: TAS_1, TAS_3, TAS_6, TAS_9, TAS_11, TAS_13, TAS_14), Difficulty Describing Feelings (DDF: TAS_2, TAS_4, TAS_7, TAS_12, TAS_17), and the reduced Externally Oriented Thinking (EOT: TAS_10, TAS_15, TAS_19) factors. The model was estimated using the WLSMV estimator suitable for ordinal data. Model fit indices indicated acceptable to good fit: CFI = .991, TLI = .989, RMSEA = .072, and SRMR = .059 (robust CFI = .958, robust TLI = .949, robust RMSEA = .094), with 87 degrees of freedom. Standardized factor loadings for all items were moderate to strong (DIF: .54–.90; DDF: .61–.91; EOT: .68–.75). Latent factors were significantly and positively correlated (r = .42–.86). These results support the adequacy of the three-factor structure with a reduced EOT subscale, although the RMSEA slightly exceeds the conventional cutoff for good fit, suggesting some model misfit may remain.

4. **Comparative Psychometric Evaluation:**

Although both models demonstrated broadly acceptable fit, the reduced EOT model provided slightly superior fit indices. Specifically, the reduced EOT model exhibited a Comparative Fit Index (CFI) of .991, Tucker-Lewis Index (TLI) of .989, Root Mean Square Error of Approximation (RMSEA) of .072, and Standardized Root Mean Square Residual (SRMR) of .059. In contrast, the original TAS-20 model yielded a CFI of .984, TLI of .981, RMSEA of .074, and SRMR of .067.

Supplementary tables

Table S1 Pairwise comparisons of study variables by Gender with Bonferroni-Adjusted Wilcoxon Tests and Effect Sizes

Variable	Gender Categories		Statistic	Effect size	p (Bonferroni adjustment)
DDF	Male	Female	8702.5	0.03	1.000
	Male	Non-binary	970.5	0.24	0.039
	Female	Non-binary	5385.5	0.20	0.001
DIF	Male	Female	8644.5	0.02	1.000
	Male	Non-binary	886.5	0.30	0.007
	Female	Non-binary	5302.5	0.20	0.000
EOT	Male	Female	10245	0.14	0.020
	Male	Non-binary	1536.5	0.12	0.678
	Female	Non-binary	7219.5	0.06	0.768
Disorganized Schizotypy	Male	Female	9511	0.09	0.280
	Male	Non-binary	896.5	0.29	0.009
	Female	Non-binary	4221	0.29	0.000
Positive Schizotypy	Male	Female	9652	0.10	0.176
	Male	Non-binary	1124.5	0.15	0.414
	Female	Non-binary	5540.5	0.19	0.001

Table S2 Estimated edge weights of the network model

Node1	Node2	Weight
depression	Negative schizotypy	0.2
Describing feelings	Negative schizotypy	0.2
Externally Oriented Thinking	Negative schizotypy	0.2
Disorganized schizotypy	Positive schizotypy	0.31
Anxiety	Positive schizotypy	0.14
Identifying feelings	Positive schizotypy	0.16
Stress	Disorganized schizotypy	0.16
Identifying feelings	Disorganized schizotypy	0.24
anxiety	Stress	0.34
Depression	Stress	0.27
Identifying feelings	Stress	0.23
Depression	Anxiety	0.27
Identifying feelings	Anxiety	0.16
Identifying feelings	Describing feelings	0.46
Externally Oriented Thinking	Describing feelings	0.33

Table S3. Strength and bridge centrality metrics

	**Strength**	**Bridge**
Negative schizotypy	0.59	0.59
Positive schizotypy	0.61	0.30
Disorganized schizotypy	0.71	0.40
Aberrant salience	1.00	0.39
Stress	0.91	0.29
Anxiety	0.74	0.20
Depression	0.99	0.20
Describing feelings	1.25	0.79
Identifying feelings	0.53	0.20
Externally Oriented Thinking	0.59	0.59

Supplementary Figures

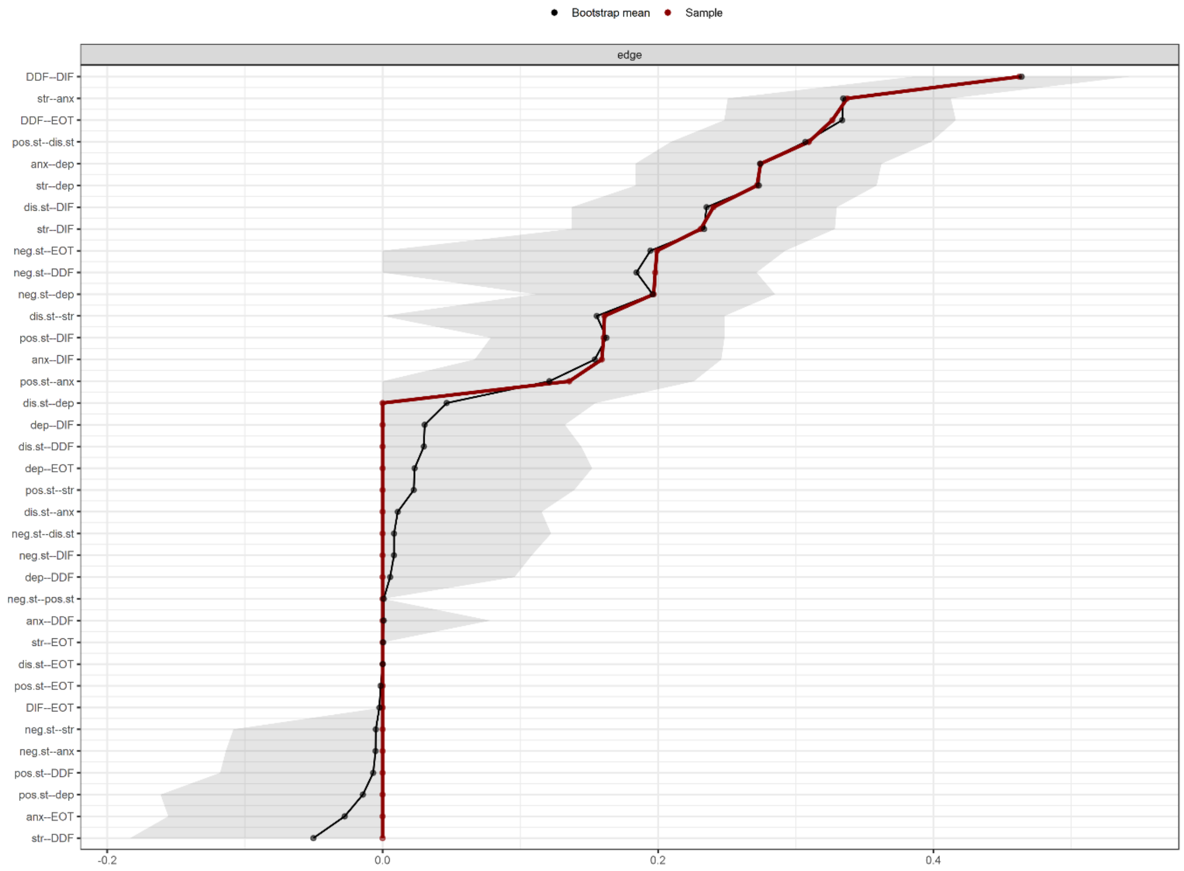

Figure S1. Edge weight accuracy based on case-drop bootstrapping
